# After the Vacation

**Published:** 1921-11

**Authors:** C. N. Johnson


### AFTER THE VACATION

Vacations are the breathing spots of a panting humanity. We go at such a rapid pace these days that we would fall exhausted if we did not let up at times, and give the straining nerves, and muscles, and blood vessels a chance to relax. The best description of a vacation I ever heard was from a gentleman of large interests who had been away from home for a few weeks, and when asked where he had gone, he said: "I have been down to the seashore to *unwind.*" That is precisely the process when we go away on the right kind of a vacation. During the rest of the year we are so incessantly occupied with the high-tension duties of the day that we get keyed up to a pitch that is abnormal, and unless we unwind occasionally, and let the machinery recover from the stress, something will eventually break.

But the hard part of a vacation is immediately after. One would think that the rested tissues would leap to their work with a vim and zip that would make the heaped-up duties melt away, and there are some men who seem to be able to do this. But with most of us the first few days are a drag, and the machinery does not run just right. It seems to require time to get it tuned up again so it will move smoothly, and without more or less friction. For this reason one is sometimes disposed to doubt that a vacation is as beneficial as has been supposed, and yet the truth is that the chief benefit from a vacation frequently comes some time after. If we but have patience and work away faithfully, the rhythm will come eventually, and we will acquire a zest for our work which makes it a real pleasure. And oh, the joy of doing things when we have once "hit our stride," and are able to make every move count. There is no satisfaction in life quite equal to that of being able to add something of real value to the sum total of human achievement each day, to see men made more comfortable and happier as the result of our ministrations, and to feel that we are an integral part of the great human machinery that makes the world a better place in which to live.

To be a nonentity in life would seem the worst calamity that could befall a man, and yet no one need be a nonentity—however limited his ability may be. Every man is given sufficient talent, if he only applies it to the best advantage, to accomplish something of merit in the world. It may be only to build the best mousetrap, or to make a cubic yard of earth yield more than some other cubic yard. No matter what it is, if he accomplishes something for the welfare of the race he is no longer a nonentity.

It may require more application in one case than in another, and yet with few men does achievement come without application. Long-continued hard work is the surest road to accomplishment of any kind, and the reason men are working so incessantly today is because they have learned this fundamental fact. The geniuses who can arrive at results by the short cut are very rare. Few things are done by the insipirational method—most are done by patient plodding—otherwise plodding would not be the order of the hour. There are more men relatively to our population who are working hard today than ever before—more men who apply themselves incessantly to the task in hand, and, therefore, more is being accomplished than in any age since the history of man began.

This is why men have to stop working at times to catch their breath—why the vacation has become an established institution in the land. We achieve more in the ultimate because we take vacations, and store up the energy whch enables us to run at top speed in the intervals. Let us not be discouraged if, when we return from a vacation, we do not at once strike our accustomed gait and make the pace we have set for ourselves. The pace will come if we patiently work for it, and when it does come we shall be able to maintain it better because of having taken the rest and renewed our energy. A vacation is not a mistake—it is a necessity.—C. N. Johnson, in *Oral Health.*

